# Correlation Between Microstructural Evolution and Magnetocaloric Response in Suction-Cast MnCoGeB_0.02_ Alloy

**DOI:** 10.3390/ma19061144

**Published:** 2026-03-15

**Authors:** Rafael Suárez, Israel Betancourt, Jesús Arenas, Marco Camacho, Israel Núñez-Tapia, Jonathan Zamora

**Affiliations:** 1 División de Ciencias Básicas, Facultad de Ingeniería, Universidad Nacional Autónoma de México, Ciudad de México 04510, Mexico; 2 Instituto de Investigaciones en Materiales, Universidad Nacional Autónoma de México, Ciudad de México 04510, Mexico; 3 Instituto de Física, Universidad Nacional Autónoma de México, Ciudad de México 04510, Mexico

**Keywords:** suction casting, microstructure, magnetocaloric effect

## Abstract

Magnetic and structural transitions can interact significantly, leading to an enhanced magnetocaloric effect (MCE), also known as the giant or colossal effect. In this study, we investigate how subtle microstructural changes impact the magnetocaloric behavior of a MnCoGeB_0.02_ alloy fabricated via suction casting. We obtained conical samples and analyzed them to understand their structure and magnetic properties. X-ray diffraction patterns revealed a coexistence of a metastable high-temperature hexagonal phase and a stable low-temperature orthorhombic phase in different regions of each cone. The presence and proportion of these phases determine the degree of magneto-structural coupling, which in turn influences the MCE. The magnetic entropy change (**|**Δ*S*_Peak_**|**) varied notably among the samples, ranging from 12.3 to 6 Jkg^−1^K^−1^ under a magnetic field change of Δµ_0_*H* = 5.0 T. These findings demonstrate that even minor microstructural changes caused by differences in solidification during suction casting can lead to noticeable variations in magnetocaloric performance. Understanding and controlling these microstructural details is vital for optimizing the functional behavior of MnCoGe-based materials.

## 1. Introduction

Humanity faces an energy crisis, paradoxically driven by the technological progress that characterizes our era. In this critical moment, the main challenge is clear: transitioning to clean energy is not optional but an existential necessity. To address this, researchers are leading the development of innovative technologies. They are investigating various energy sources and advanced methods to achieve sustainability, aiming to optimize resource use and develop next-generation energy systems.

Refrigeration and air conditioning account for a significant portion of electrical energy consumption in both residential and commercial sectors [1]. The magnetocaloric effect (MCE) represents a promising approach for supplementing or replacing conventional gas-compression technologies through magnetic refrigeration (MR). MR offers relevant advantages as a green technology, mainly enhanced energy efficiency and the avoidance of chlorofluorocarbon-based refrigerants.

The MCE is a phenomenon where a material undergoes a reversible temperature change when an external magnetic field is applied or removed. This thermal response results from changes in magnetic order, which is the alignment or random ordering of magnetic moments within the material. From a thermodynamic perspective, the MCE occurs due to variations in the total entropy (*S*_T_) of the system induced by variations in the applied magnetic field. Total entropy is the sum of lattice entropy (*S*_l_), electron entropy (*S*_e_), and magnetic entropy (*S*_m_). When a magnetic field is applied under adiabatic conditions, magnetic moments tend to align, causing a decrease in *S*_m_. To maintain constant *S*_T_, this decrease is balanced by an increase in lattice and electronic entropies, leading to a measurable rise in temperature. Conversely, removing the magnetic field causes magnetic disordering, which increases *S*_m_ and results in an adiabatic cooling effect.

A particularly strong MCE is observed in materials exhibiting coupled magnetic and structural transitions (*T*_S_). When these transitions are coupled, the phenomenon is called a magneto-structural transition, often producing a giant entropy change. A well-known example is Gd_5_Si_2_Ge_2_, where Pecharsky and Gschneidner reported |Δ*S*_m_| of ~14 JKg^−1^K^−1^ and Δ*T*_ad_ ~6 K for Δµ_0_*H* = 2.0 T due to a first-order magneto-structural transition [2]. This discovery established the importance of coupling structural and magnetic degrees of freedom to enhance MCE performance.

Comprehensive reviews by Franco et al. [3,4] classified magnetocaloric materials based on their structure (amorphous and crystalline) and composition (rare-earth-based or non-rare-earth-based). They emphasized the importance of optimizing the relationship between the *T*_S_ temperature and the Curie temperature (*T*_C_). Among rare-earth materials, gadolinium (Gd) remains a standard for room-temperature (298 K) refrigeration due to its second-order magnetic transition around 294 K, and a moderate entropy change (~5.54 Jkg^−1^K^−1^ at 2.0 T) [5,6]. However, the high cost and limited availability of rare-earth elements drive the search for alternative systems.

In this context, manganese-based compounds have attracted considerable interest due to their lower cost and compositional versatility. These include Laves phases, MnAs alloys, Heusler alloys, Mn(Fe, Co, Ni)(Si, Ge), (Mn, Fe, Co)_5_(Ge, Si, Sb)_3_, MnFe(P, As) alloys, perovskites [3,4,7], and high-entropy alloys [8,9,10]. Among these, Mn(Co, Fe, Ni)(Ge, Si)-based alloys are especially attractive because their magneto-structural properties can be tuned through chemical variations or by applying hydrostatic pressure [11,12,13,14,15].

MnCoGe undergoes a martensitic transition from a low-temperature orthorhombic TiNiSi-type structure (space group *Pnma*) to a high-temperature hexagonal Ni_2_In-type structure (space group *P63*/*mmc*) upon heating. These phases exhibit different magnetic behaviors due to variations in Mn-Mn interactions. For instance, the orthorhombic phase shows a magnetic saturation (*M*_s_) of 4.13 µB and *T*_C_ = 345 K, while the hexagonal phase presents lower values, with *M*_s_ and *T*_C_ of 2.76 µB and 265 K, respectively [16]. Numerous studies have demonstrated that compositional modifications (such as substitution or vacancy) and heat treatments can shift *T*_S_ and *T*_C_ to improve the overlap of magneto-structural transitions and enhance |Δ*S*_m_| [11,12,13,14,15].

Although these advances are significant, most studies mainly explore chemical adjustments or thermal processing as control methods. The role of geometry-related solidification kinetics and microstructural gradients within a single alloy composition has been less thoroughly examined. Specifically, the influence of different cooling rates on phase fractions, crystallographic orientations, and magneto-structural coupling in MnCoGe-based systems remains underexplored.

This study addresses this gap by investigating the MnCoGeB_0.02_ alloy fabricated in a conical geometry via suction casting. The inherent cooling-rate gradient along the cone axis enables controlled microstructural changes without altering the composition or heat-treatment process. Boron, which occupies interstitial sites in the lattice [17,18], also affects the structural and magnetic transition temperatures. By sectioning the cone into three parts: base, middle, and tip segments, it was evaluated how phase coexistence, crystallographic changes, and the relative positions of *T*_S_ and *T*_C_ influence magnetocaloric performance. This approach clarifies the processing–structure–property relationship and demonstrates that geometry-controlled solidification can complement compositional tuning strategies in magnetocaloric materials.

## 2. Materials and Methods

### 2.1. Samples Fabrication

Polycrystalline ingots of the MnCoGeB_0.02_ alloy were synthesized via arc-melting stoichiometric amounts of high-purity (Sigma-Aldrich, St. Louis, MO, USA. 99.99%) constituent elements under an argon atmosphere, using a three-purge cycle to ensure the furnace chamber was free of oxygen. To ensure sample homogeneity, the ingots were remelted four times using an Edmund Bühler GmbH model MAM-1 (Bodelshausen, Germany) arc melter, reaching a pressure of 1.9 × 10^−2^ Torr. Finally, the homogenized ingots were cast into a cone shape using a suction-casting accessory attached to the furnace system under a vacuum of 3.0 × 10^−2^ Torr.

**Figure 1:**
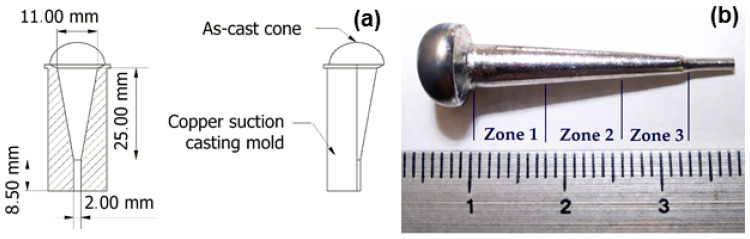
(**a**) Technical schematic of the copper suction casting mold and the resulting as-cast conical sample, shown as a longitudinal cross-sectional view along with frontal and lateral projections, including the main geometric dimensions. (**b**) Photograph of the fabricated MnCoGeB_0.02_ cone, displaying the three analyzed sections along the axial direction: Zone 1 (base), Zone 2 (middle), and Zone 3 (tip), defined by the cooling-rate gradient during solidification.

After casting, the cone was sectioned into three parts using a diamond-coated disc mounted on an Extec LabCut 150 equipment (Enfield, CT, USA). The cone was divided into three regions according to its geometry and expected cooling-rate gradient: Zone 1 (SZ1), corresponding to the widest base; Zone 2 (SZ2), located in the middle section; and Zone 3 (SZ3), corresponding to the narrowest tip. This segmentation allows a systematic evaluation of microstructural and magnetic variations induced by solidification kinetics. A schematic representation of the mold and cone geometry, together with the defined sections, is presented in Figure 1.

Subsequently, the cut specimens were annealed in a vacuum-sealed quartz ampoule (≈10^−3^ Torr) at 1123 K for 120 h to promote compositional uniformity and stabilize the equilibrium phase. Finally, the ampoules were quenched in ice water. The structural and magnetic properties of heat-treated samples were then determined.

### 2.2. Microstructural Characterization

Scanning electron microscopy studies were carried out using a JEOL JCM-6000 microscope (Tokio, Japan). Chemical composition was determined using Energy Dispersive X-ray Spectroscopy (EDS) (Tokio, Japan) spectra. The images were post-processed using the open-source software ImageJ (V1.54r) to produce high-contrast images.

The presence of crystalline phases was verified by powder X-ray diffraction analysis at room-temperature, conducted using a Bruker D8 diffractometer (Karlsruhe, Germany) with Cu Kα radiation (λ = 1.5406 Å) and the powder diffraction technique; the scan range was 20° to 80° with a step size of 0.01°. The powder was obtained by grinding the samples in an agate mortar.

Rietveld refinement was performed using the free software FullProf (V8.20), and χ^2^ > 6 was used as a parameter of adequate fit. Mathematical fitting of diffraction peaks was developed using the Thompson–Cox–Hastings (TCH) approximation.

The structural transition temperature was determined by differential scanning calorimetry (DSC). Measurements were performed using a TA Instruments Q200 DSC (New Castle, DE, USA) at a heating rate of 10 K/min from 280 K to 380 K. Data were used to calculate enthalpy transformation (H_Tr_) using Equation (1) [19].

(1) $$H_{Tr}=\int \frac{\overset{\cdot }{q}}{\beta }dt$$

### 2.3. Magnetic Characterization

Magnetic measurements were conducted using a Quantum Design MPMS3 system (San Diego, CA, USA) equipped with the VSM option. The temperature dependence of magnetization (*M-T*) was measured in the range of 200–400 K under an applied magnetic field of 50 mT, using a temperature sweep rate of 1 K/min. Isothermal magnetization curves (*M-H*) were recorded in magnetic fields ranging from 0 to 5.0 T.

The isotherms were measured between 200 K and 400 K, with temperature intervals of 5 K between successive measurements.

Magnetocaloric performance indicators were then estimated; for |Δ*S*_m_(*T*)| curves, predictions were made using the Maxwell relation (Equation (2)) [3,4]. The calculations were performed for μ_0_Δ*H* = 5.0 T.

(2) $${\left(\frac{\partial S_{m}}{\partial H}\right)}_{p,T}=\mu_{0}{\left(\frac{\partial M}{\partial T}\right)}_{p,H}$$

Additionally, refrigerant capacity (*RC*) was estimated by employing the area under the |Δ*S*_m_(*T*)| curve [3,4,20,21] as Equation (3) indicates.

(3) $$RC=\int_{T_{Cold}}^{T_{Hot}}{\Delta S}_{m}\left(T,H\right)dt$$

## 3. Results

### 3.1. Microstructural Analysis

**Figure 2:**
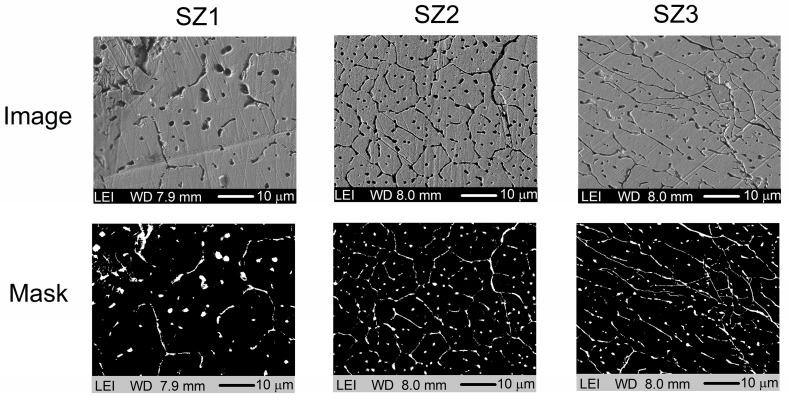
Composite SEM images of samples SZ1, SZ2, and SZ3 obtained using low-energy secondary electron detectors. The corresponding high-contrast images are shown in the lower row.

Figure 2 displays a set of SEM micrographs and their corresponding high-contrast versions. Qualitatively, partial grain-boundary reveals can be observed, with their morphology varying across samples. It is also evident that the cooling conditions change as the geometry progresses toward the narrower region, which in turn influences the solidification process. The images for sample SZ1 show the largest grain size, which decreases in sample SZ2. Finally, sample SZ3 exhibits a change in grain morphology, showing elongated cellular structures indicative of directional grain growth, attributed to the increased cooling rate near the tip.

The thermal gradient results from changes in sample geometry, promoting a transition from coarse, equiaxed to a more refined and oriented solidification structure. The preferred grain alignment, associated with a higher cooling rate, affects crystallographic orientation and is likely to lead to texture development.

Furthermore, the SEM micrographs and their high-contrast masks suggest the presence of residual-stress relaxation mechanisms associated with non-uniform solidification conditions. The observed network of partly continuous intergranular features, especially in samples SZ2 and SZ3, aligns with microcracking and localized decohesion along grain boundaries, which typically serve as pathways for stress release during thermal contraction. In SZ3, the orientation of elongated cellular structures and defect traces further supports the development of anisotropic stresses and their subsequent directional relaxation.

**Figure 3:**
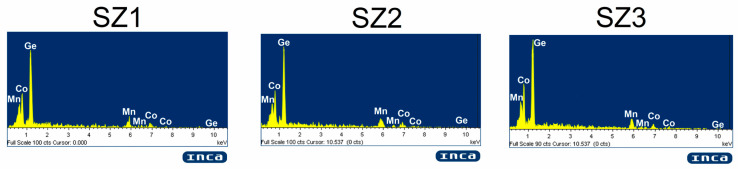
Energy Dispersive Spectroscopy spectra of samples SZ1, SZ2, and SZ3.

The nominal composition of the MnCoGeB_0.02_ alloy is approximately an atomic ratio of 33.1:33.1:33.1:0.7. The compositional analysis of samples SZ1, SZ2, and SZ3, based on the EDS data summarized in Table 1, confirms good agreement with the estimated stoichiometry. It should be noted that EDS is a semi-quantitative technique; therefore, the boron content was not directly detected in the spectra (Figure 3) due to its low atomic number and the method’s limited sensitivity to light elements.

**Figure 4:**
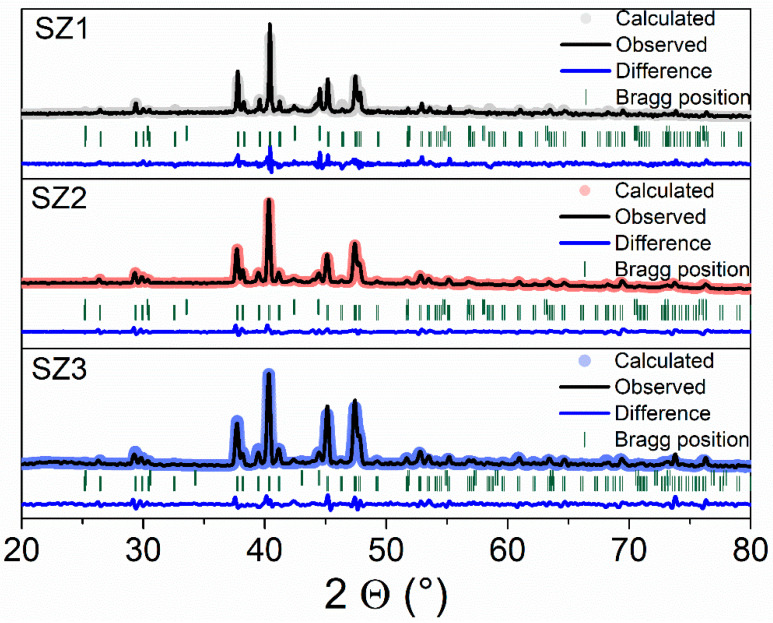
X-ray diffraction pattern and Rietveld refinement for cone section.

Phase distributions in the heat-treated samples were analyzed by X-ray diffraction patterns at room temperature. This analysis allowed for the identification of two phases: (1) the low-temperature orthorhombic TiNiSi-type structure (space group *Pnma*) and (2) high-temperature hexagonal Ni_2_In-type structure (space group *P63/mmc*). Both phases were identified using ICDD files 01-077-7496 and 01-071-4267. X-ray patterns and Rietveld refinement fits are presented in Figure 4.

Rietveld refinement statistical parameters indicate reliable and physically consistent adjustments; a summary is presented in Table 2. Refinements of the samples show excellent correlation between observed and calculated profiles [22,23,24,25]. R_wp_ values are below 10, which is considered acceptable. At the same time, R_p_ reaches a maximum of 3.49, consistent with the expected relation R_p_ < R_wp_. χ^2^ is a common parameter used to evaluate goodness of fit; the ideal value is χ^2^ ≈ 1. In this context, a χ^2^ value greater than one does not necessarily indicate an inadequate fit, as some authors suggest [26]. The sample SZ1 exhibits the highest χ^2^ value, mainly due to its lower R_exp_ value. This makes the refinement statistics fit more demanding.

Rietveld Refinement results reveal a progressive increase in the proportion of the low-temperature orthorhombic phase along the cone’s length, rising from 76.56% in SZ1 (widest section) to 92.91% in SZ3 (narrowest section). This corresponds to a steady axial gradient exceeding 10% in the crystalline phase fraction. Meanwhile, the volume of the low-temperature phase increases slightly, while that of the high-temperature phase decreases. The refined volumes for both phases remain close to their reference values for stoichiometric MnCoGe (75.69 Å^3^ and 160.29 Å^3^ from ICDD files 01-077-7496 and 01-071-4267, respectively), deviating by less than 1.8%.

Phase fraction uncertainties were derived from the standard deviations provided by the FullProf Rietveld refinement. The largest deviation was ±0.38%. Since the phase fraction varies by more than 16% along the cone, the observed differences significantly surpass the refinement uncertainties and are therefore statistically meaningful.

**Figure 5:**
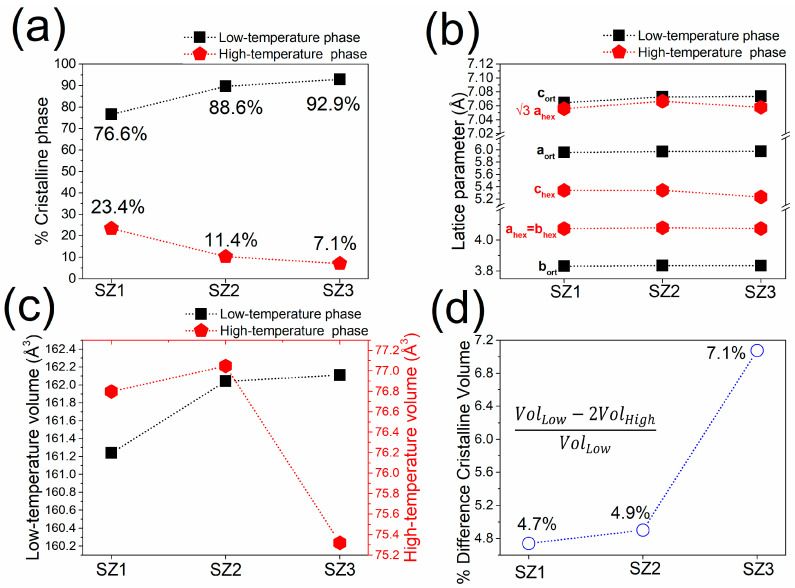
Refinement data for the percent crystalline phases (**a**), lattice parameter (**b**), volume phase of the high- and low-temperature phases (**c**), and percent volume difference between crystalline phases (**d**). Data correspond to the three cone zones, the dotted lines are only intended as visual guide and do not represent a statistical fit.

On the other hand, Figure 5a shows a steady increase in the orthorhombic phase fraction from SZ1 to SZ3, confirming that the cooling-rate gradient caused by the cone shape influences phase stability along the axial direction. Because the orthorhombic phase is linked with stronger magnetic ordering, its gradual stabilization directly impacts the magneto-structural balance of the alloy. Figure 5b shows subtle but consistent variations in lattice parameters, reflecting changes in internal strain and Mn–Mn interatomic distances. In MnCoGe-based systems, magnetic exchange interactions are very sensitive to lattice distortions, so these structural changes are particularly important for the magnetic transition.

The unit cell volumes in Figure 5c generally increase toward the narrower section; however, SZ3 deviates from this pattern, showing a reduction in the high-temperature phase volume. Microstructural analysis from TCH Rietveld refinement reveals that maximum lattice macrostrain values (>9 × 10^−3^) coexist with large coherent crystalline domains. This implies that in SZ3, the decrease in the high-temperature phase volume results from better internal strain relaxation and reduced microdeformation under higher cooling rates.

Lastly, Figure 5d depicts a phase volume difference ranging from 4.74% to 7.07%, with SZ3 exhibiting the largest mismatch. This volumetric discrepancy affects the overlap between structural (*T*_S_) and magnetic (*T*_C_) transitions, thereby influencing both the strength and distribution of the magnetocaloric response.

**Figure 6:**
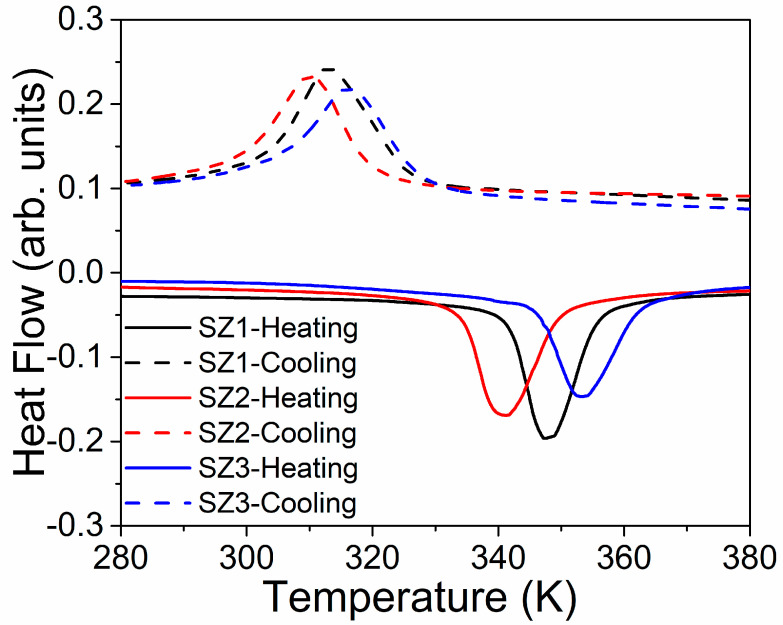
Differential Scanning Calorimeter heating and cooling curves are shown as solid and dashed lines, respectively.

Structural transition temperatures during heating and cooling are shown in Figure 6, which plots displacement during the transition as a function of the zone. During heating, *T_S_* were detected at 348 K, 341 K, and 354 K for Zones 1, 2, and 3, respectively. Meanwhile, during cooling, *T*_S_ were observed at 313 K (Zone 1), 309 K (Zone 2), and 315 K (Zone 3). The structural transition varies by approximately 11 K and 6 K during heating and cooling, respectively.

We assumed that the phase fraction and cell volume variations are related to differences in structural transition. Various studies show a correlation between high-temperature volume-phase changes and the structural transition temperature [27,28,29]. The present data indicate that larger unit cell volumes are associated with lower activation energies for structural transitions. The high-temperature volume phase and the corresponding structural transition temperatures obtained are consistent with other reports in the literature [30]. Meanwhile, the fraction of the high-temperature phase is related to the transition enthalpy required to drive the structural phase transformation.

H_Tr_ estimates from Figure 6 are included in Table 3. The data show slightly higher values during cooling than during heating, with differences of 15% to 12%, indicating first-order transformation thermodynamics. The trend in the H_Tr_ values shows a decreasing pattern, which aligns with the previous Rietveld refinement results.

### 3.2. Magnetic Transition

**Figure 7:**
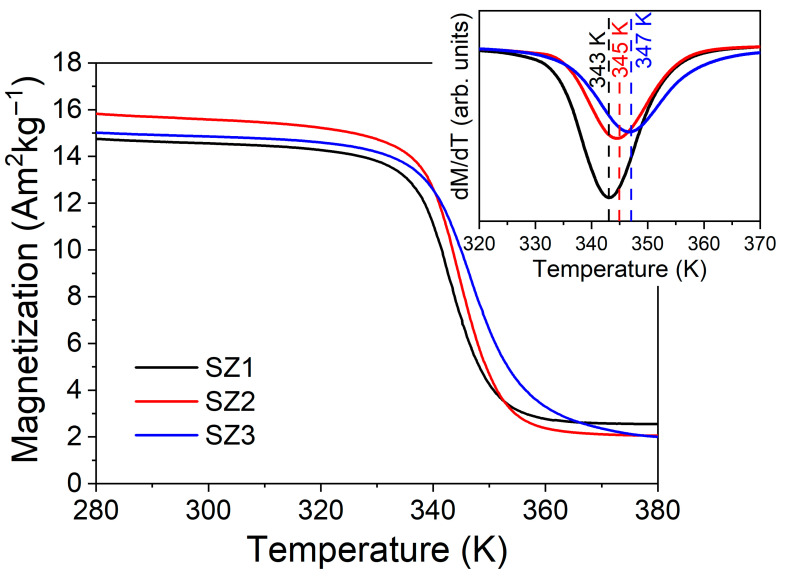
Magnetization–Temperature comparison for each sample, as well as the derivative indicating *T*_C_. The inset indicates that SZ1 (black line) shows the most abrupt variation, i.e., the smallest and largest derivative magnitudes, followed by SZ2 (red line). Finally, SZ3 (blue line) has a magnitude similar to SZ2 but a larger temperature range. For details about the color references in this figure legend, please refer to the online version of this article.

Figure 7 shows the thermomagnetic curves detected at the *T_C_* temperature. *T*_C_ was observed at 343 K ± 0.1 K for SZ1, 345 K ± 0.1 K for SZ2, and 347 K ± 0.3 K for SZ3. The experimental results show slight variations in the magnetic transitions and magnetization during the magnetic order-to-disorder process, with a small shift in *T*_C_. The d*M*/d*T*(*T*) curves are shown in the inset; the magnitude of the transitions and their *T*_C_ values are related to the percentage of the high-temperature phase.

High-temperature crystalline structures exhibit the lowest *T_C_*, with values reported to range from 260 to 283 K in similar alloys. Meanwhile, low-temperature phase transitions occur at approximately 355 K [16,29,31,32,33]. As the percentage of the high-temperature phase increases, transitions in the sample occur at lower temperatures; this trend has been observed in previous studies [33]. These results confirm the coexistence and relative fractions of low- and high-temperature phases across the cone section, as estimated by Rietveld refinement.

### 3.3. Structural and Magnetic Transition Comparison

The *M-T* curves showed a similar *T_C_* for the material, with a maximum variation of 4 K across the collected data. In contrast, DSC analysis revealed structural transition variations exceeding 12 K.

**Figure 8:**
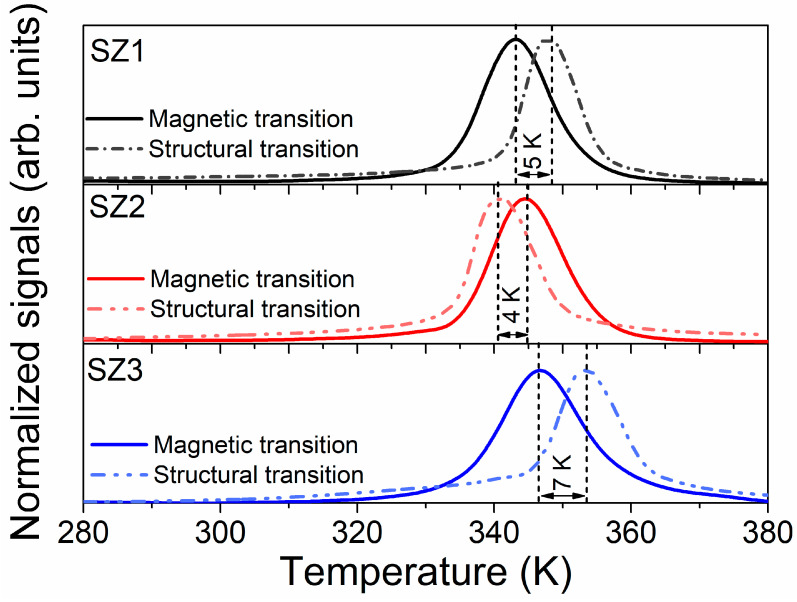
Comparison of magnetic and structural transitions for each zone, along with temperatures observed during heating transitions. The figure compares DSC data with the normalized absolute derivative of the Magnetization–Temperature curve for cone samples; both signals are normalized.

An assessment of normalized structural and magnetic transitions is presented in Figure 8. This comparison provides important insights into the magneto-structural transformations that are coupled with MCE performance. The gap calculated between the structural and magnetic transformations (|*T_S_* − *T_C_*|) was 7 K in SZ3, 5 K in SZ1, and 4 K in SZ2. The order of the *T_S_* and *T_C_* temperatures is reversed in the SZ2 sample, where the structural transition occurs before the magnetic transitions. In contrast, the other samples exhibit an inverted sequence of magnetic and structural transitions. Additionally, the structural transition in SZ3 starts near the end of the magnetic transition.

### 3.4. Magnetocaloric Performance

**Figure 9:**
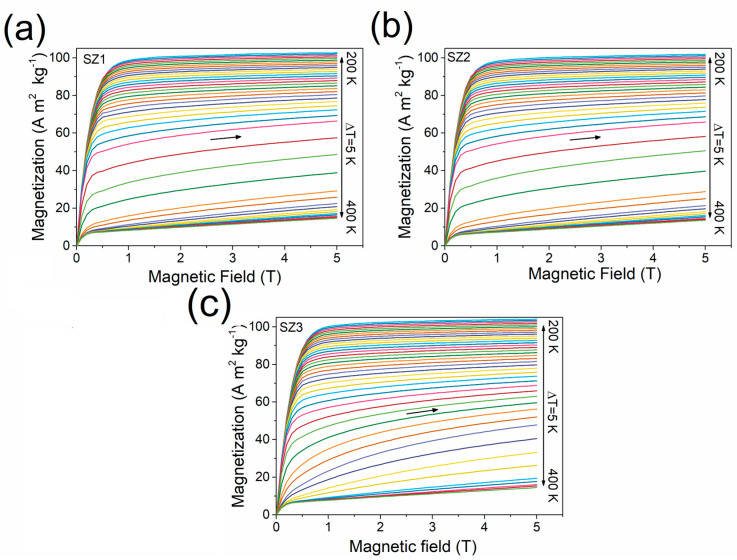
Magnetization isotherm curves *M*(*H*) for cone segment samples SZ1 (**a**), SZ2 (**b**), and SZ3 (**c**), measured under magnetic fields ranging from 0 to 5.0 T. The isotherms were recorded over the temperature range from 200 to 400 K, with a temperature step of 5 K (Δ*T* = 5 K). A color scale is used to differentiate each isotherm, as indicated in the legend. Measurements were carried out under an increasing magnetic field, as indicated by the horizontal arrows in the plots.

The isothermal magnetization curves shown in Figure 9 display the magnetic response of SZ1 (a), SZ2 (b), and SZ3 (c) under applied fields ranging from 0 to 5.0 T in the temperature range of 200–400 K, with 5 K intervals. These curves serve as the experimental basis for calculating the magnetic entropy change |Δ*S*_m_(*T*)| using the Maxwell relation (Equation (2)), since |Δ*S*_m_| depends directly on the temperature derivative of magnetization with respect to the applied magnetic field. The slope of the *M*-*H* curves changes with increasing temperature. At lower temperatures (around 200 K), the curves exhibit rapid magnetization saturation at relatively low fields, typical of a ferromagnetic state. In contrast, at higher temperatures (near 400 K), the magnetization increases more gradually with the field, indicating the paramagnetic state. The gradual decrease in slope near the transition region indicates the magnetic order–disorder transformation.

In SZ1 and SZ2 (Figure 9a,b), a more pronounced separation between consecutive isotherms is observed near the transition temperature. This behavior suggests stronger coupling between magnetic and structural transitions, thereby enhancing the entropy change. In contrast, SZ3 (Figure 9c) exhibits a smoother, more distributed evolution of the isotherms, consistent with a broader transition and a reduced peak |Δ*S*_m_|, as discussed previously.

The magnetic entropy change (|Δ*S*_m_|) was calculated from isothermal *M-H* curves using the Maxwell relation (Equation (2)) under strictly controlled experimental conditions, with temperature stability within ±0.1 K and 5 K temperature intervals. To minimize experimental uncertainties and ensure reproducibility, the measurement protocol described in reference [34] was carefully followed, limiting instrumental errors to below 1% in the magnetocaloric measurements.

**Figure 10:**
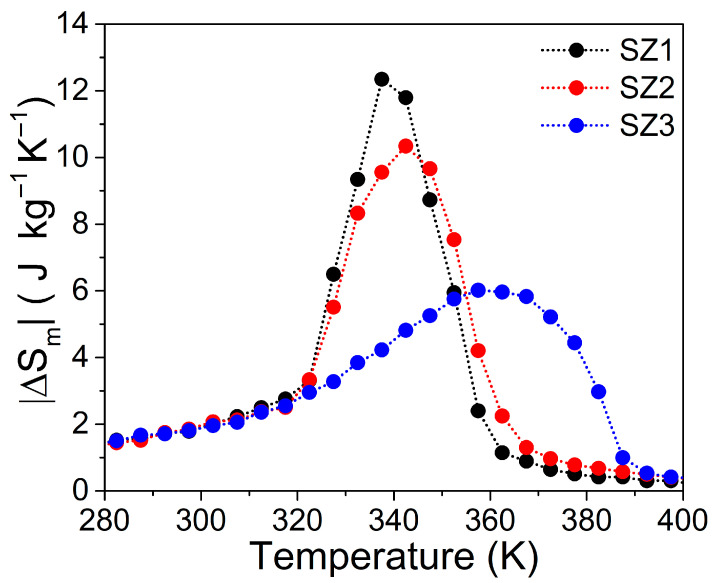
Temperature dependence of the magnetic entropy change |Δ*S*_m_| for samples SZ1, SZ2, and SZ3 under a magnetic field change of μ_0_Δ*H* = 5.0 T. The curves were obtained via Maxwell integration of *M*-*H* data, following the protocol described in reference [34].

The MCE, as shown in the |Δ*S*_m_(*T*)| curves (Figure 10), exhibited maximum fluctuations over a 20 K range (from *T*_cold_ to *T*_hot_) between samples. The |Δ*S*_m_(*T*)| performance shows significant variations in Δ*S*_Peak_ and *T*_Peak_ for different sections of the cone. *T*_Peak_ ranges from 337.5 K to 357.5 K; while Δ*S*_Peak_ varies from 12.3 Jkg^−1^K^−1^ to 6 Jkg^−1^K^−1^. Both datasets show a consistent trend across cone samples, with MCE decreasing as the section decreases. A notable asymmetry is evident in the SZ3 curve, likely due to the temperature difference between *T_C_* and *T_S_*.

## 4. Discussion

Table 4 summarizes magnetocaloric data for MnCoGe-based alloys, with maximum magnetic entropy changes ranging from 4 to 27.8 Jkg^−1^K^−1^ and peak temperatures (*T*_Peak_) from 279 to 360 K. The current samples (SZ1, SZ2, SZ3) show intermediate |Δ*S*_Peak_| values between 6 and 12.3 Jkg^−1^K^−1^. Although these are lower than those achieved through compositional tuning (e.g., MnCo_0.94_Ge_0.06_, which reaches about 27.8 Jkg^−1^K^−1^ within a narrow 7 K window), the samples exhibit significantly broader operating temperature ranges (around 23–47 K). For practical room-temperature magnetic refrigeration, an ideal material balances |Δ*S*_Peak_|, *T*_Peak_, and *T*_hot_-*T*_cold_. This study shows that geometry-controlled processing can attain this balance through magneto-structural control rather than by altering composition.

The *RC* of the samples ranges from 231 Jkg^−1^ to 247 Jkg^−1^, indicating similar values among them. This similarity in behavior can be attributed to the balance between the parameters |Δ*S*_Peak_| and *T*_hot_-*T*_cold_. Although SZ3 has the lowest entropy peak, its wider operating temperature range offsets this decrease, leading to the highest refrigerant capacity.

The differences in MCE among the cone sections primarily stem from microstructural variations caused by the cooling-rate gradient. Although the Curie temperature shifts slightly (from 343 to 347 K), small changes in magnetization and transition sharpness are observed. Similar sensitivities to structural changes have been reported in MnCoGe systems with element substitution or heat treatment adjustments [14,27,37,38]. In this case, these differences arise solely from geometry-driven solidification effects.

Rietveld refinement indicates that the volume fraction of low- and high-temperature phases varies along the cone, with the orthorhombic phase increasing from 76.56% to 92.91%. This phase redistribution, driven by solidification kinetics, directly impacts magneto-structural coupling. The samples exhibit |*T*_S_ − *T*_C_| values ≥ 5 K, indicating coupled structural and magnetic transitions. The relative order of *T*_S_ and *T*_C_ significantly influences the magnitude and symmetry of the entropy peak.

Microstructural analysis reveals a transition from coarse, equiaxed grains in SZ1 to finer, elongated cells in SZ3, indicating the development of crystallographic texture. Since magnetic exchange interactions in MnCoGe alloys are highly sensitive to lattice symmetry and Mn–Mn spacing, preferred orientation and strain redistribution can broaden the transition and modify entropy behavior. This explains why SZ1 shows the highest |Δ*S*_Peak_| (12.3 Jkg^−1^K^−1^), while SZ3 exhibits a lower but broader response, resulting in a higher refrigerant capacity of 247.51 Jkg^−1^.

The results demonstrate a clear structure–orientation–property relationship: the cooling-rate gradient modifies phase fraction, strain state, and likely crystallographic orientation; these factors alter the overlap and sequence of structural and magnetic transitions; and this interplay ultimately determines whether the magnetocaloric response is sharp and intense or broader and moderate. Thus, geometry-induced microstructural control emerges as a viable and complementary strategy for tuning magnetocaloric performance in MnCoGeB_0.02_.

## 5. Conclusions

In summary, the experimental results from the structural, microstructural, thermal, and magnetic characterization of the suction-cast MnCoGeB_0.02_ conical samples are as follows:

- The conical shape created a measurable cooling-rate gradient along the axial direction, leading to a gradual change in the orthorhombic phase fraction from 76.56% (SZ1) to 92.91% (SZ3). This confirms that solidification kinetics strongly influence phase stability.
- Structural analysis showed phase volume differences from 4.74% to 7.07%, with subtle changes in lattice parameters. These indicate that internal strain and Mn–Mn interatomic distances are affected by the geometry-dependent solidification.
- The structural transition temperature (*T*_S_) varied by up to 13 K among sections, while the Curie temperature (*T*_C_) shifted only slightly (343–347 K). This suggests that microstructural changes primarily impact magneto-structural coupling rather than the intrinsic magnetic order.
- The magnetic entropy change |ΔSₚₑₐₖ| ranged from 12.3 Jkg^−1^K^−1^ (SZ1) to 6 Jkg^−1^K^−1^ (SZ3) under μ_0_Δ*H* = 5.0 T. The *T*_Peak_ varied by approximately 20 K, indicating that phase coexistence and transition sequence significantly influence the magnitude of the MCE.
- Samples showed |*T*_S_ − *T*_C_| ≥ 5 K, confirming coupled structural and magnetic transitions. The order and overlap of *T*_S_ and *T*_C_ control the sharpness and symmetry of the entropy peak.
- Despite differences in |ΔSₚₑₐₖ|, the refrigerant capacity (*RC*) remained within a narrow range (231–247 Jkg^−1^). This is due to a balance between the magnitude of entropy and the temperature span (*T*_hot_-*T*_cold_), especially in SZ3.
- These results reveal a clear processing–structure–property relationship: geometry-induced solidification alters phase fraction, strain state, and possibly crystallographic orientation, which in turn governs magneto-structural coupling and magnetocaloric performance.

In general, geometry-controlled solidification is a viable and complementary strategy for optimizing the magnetocaloric behavior of MnCoGe-based alloys, complementing compositional tuning.

## Figures and Tables

**Table 1:** Summary of the elemental composition ratios of the samples.

	SZ1		SZ2		SZ3	
Element	Weight %	Atomic %	Weight %	Atomic %	Weight %	Atomic %
Mn	25.37	29.28	30.7	33.64	29.7	32.71
Co	36.64	36.54	37.19	35.2	35.11	32.51
Ge	37.99	34.18	32.11	31.16	35.19	34.78

**Table 2:** Summary of statistical parameters of Rietveld refinement.

Sample	SZ1	SZ2	SZ3
χ^2^	5.7	2.37	3.37
R_wp_	4.59	4.45	4.87
R_p_	3.11	2.98	3.49
R_exp_	1.92	2.89	2.65

**Table 3:** Enthalpy transition values on heating and cooling process.

H_Tr_	SZ1	SZ2	SZ3
Cooling (Jg^−1^)	10.96	9.76	8.32
Heating (Jg^−1^)	12.57	11.19	9.8

**Table 4:** Resume of MCE performance parameters of MnCoGe-based alloys at μ_0_Δ*H* = 5.0 T.

Sample	Δ*S*_Peak_ Jkg^−1^K^−1^	*T*_Peak_ K	*T*_cold_ K	*T*_hot_ K	*T*_hot_-*T*_cold_ K	*RC* Jkg^−1^	*T_S_* K	*T_C_* K	Ref.
SZ1	12.3	337.5	327.79	350.71	22.92	231.97	348	343	This work
SZ2	10.3	342.5	327.78	354.86	27.08	237.51	341	345	This Work
SZ3	6	357.5	330.15	378.01	47.86	247.51	354	347	This Work
MnCoGe	4	~360 ^1^	314	385	71	218	-	355	[35]
MnCo_0.94_Ge_0.06_	27.8	296	293	300	7	227 ^2^	295	296	[36]
Mn_0.96_Cr_0.04_CoGe	10.6	~300 ^1^	~289 ^1^	~311 ^1^	22	185	~316 ^1^	~310 ^1^	[32]
MnCo0.96Bi_0.04_Ge	6.16	~279 ^1^	-	-		170.35	267	279	[30]
Mn_0.89_Cr_0.11_CoGe	27.7	~288 ^1^	-	-		-	-	292	[33]

^1^ Values estimated from reported curves. ^2^ *RC* value was estimated by employing $RC=\left(T_{hot}-T_{cold}\right)\Delta S_{peak}$.

## Data Availability

The original contributions presented in this study are included in the article. Further inquiries can be directed to the corresponding authors.
